# What can associative learning do for planning?

**DOI:** 10.1098/rsos.180778

**Published:** 2018-11-28

**Authors:** Johan Lind

**Affiliations:** 1 Centre for Cultural Evolution, Stockholm University, Stockholm, Sweden; 2 Department of Zoology, Stockholm University, Stockholm, Sweden

**Keywords:** planning, associative learning, reinforcement learning, animal intelligence, flexible behaviour

## Abstract

There is a new associative learning paradox. The power of associative learning for producing flexible behaviour in non-human animals is downplayed or ignored by researchers in animal cognition, whereas artificial intelligence research shows that associative learning models can beat humans in chess. One phenomenon in which associative learning often is ruled out as an explanation for animal behaviour is flexible planning. However, planning studies have been criticized and questions have been raised regarding both methodological validity and interpretations of results. Due to the power of associative learning and the uncertainty of what causes planning behaviour in non-human animals, I explored what associative learning can do for planning. A previously published sequence learning model which combines Pavlovian and instrumental conditioning was used to simulate two planning studies, namely Mulcahy & Call 2006 ‘Apes save tools for future use.’ *Science* **312**, 1038–1040 and Kabadayi & Osvath 2017 ‘Ravens parallel great apes in flexible planning for tool-use and bartering.’ *Science* **357**, 202–204. Simulations show that behaviour matching current definitions of flexible planning can emerge through associative learning. Through conditioned reinforcement, the learning model gives rise to planning behaviour by learning that a behaviour towards a current stimulus will produce high value food at a later stage; it can make decisions about future states not within current sensory scope. The simulations tracked key patterns both between and within studies. It is concluded that one cannot rule out that these studies of flexible planning in apes and corvids can be completely accounted for by associative learning. Future empirical studies of flexible planning in non-human animals can benefit from theoretical developments within artificial intelligence and animal learning.

## 1. Introduction

To the amazement of the world, associative learning models used in artificial intelligence (AI) research now achieve human level skills in video games [1] and beat human masters in the Chinese board game Go [2], chess and shogi [3]. Despite the fact that associative learning within AI research is acknowledged for producing human-like behaviour, associative learning is often either not mentioned (e.g. [4–8]), or perceived as unwanted or of insufficient sophistication (e.g. [9–14]) to provide explanations for flexible behaviour in non-human animals. It is an intriguing paradox that associative learning is acknowledged for producing complex flexible behaviour within AI research, but is often dismissed and neglected as a model for flexible behaviour in biological systems (both humans and non-human animals).

Whether the development of behaviour sequences in non-human animals can be understood in terms of associative learning or not has far-reaching consequences for our understanding of the study of behaviour. If behaviour perceived as advanced or complex, such as chimpanzee (*Pan troglodytes*) tool use, can develop through associative processes, species differences can be sought in terms of genetic differences in behaviour repertoires, exploratory tendencies such as curiosity, and motivational and attentional factors. If associative processes do not suffice to account for how information is processed and memories are updated to generate behaviour, then alternative mechanisms must be identified for us to understand how such behaviour develops. Today researchers have very contrasting views on this issue. On the one side, some suggest that associative processes, together with factors such as behaviour repertoire size and exploration are powerful and can explain a great deal of how animals acquire behaviour (e.g. [15–19]). By contrast, others emphasize alternative mechanisms and propose that animals have many different mechanisms that solve different specific problems and that these mechanisms are fine-tuned by evolution (e.g. [4,10,20]). Not all studies fall into these two categories and some studies test alternative explanations and control for associative learning. However, it is common that such studies assume only the simplest forms of associative learning. This is likely to result in false rejections of associative learning hypotheses. This is because most vertebrates and invertebrates exhibit capacities for both instrumental and Pavlovian learning [21,22], that together with specialized memories [23] make most animals capable of more complex learning than what the simplest forms of associative learning allow.

The aim of this study was to explore if a learning model [19], similar to reinforcement learning used in AI research, can help us understand the acquisition of planning behaviour in corvids and apes, behaviours sometimes perceived as complex and human-like. It has been concluded that several species plan flexibly for the future, not unlike humans (e.g. [24–28]). The idea is that this kind of planning is an outcome of a flexible mental mechanism that can simulate, mentally, different future states from current information. However, these claims have been contested based on at least two different lines of arguments. First, researchers have raised doubts concerning birds’ general capacity to plan because planning studies in birds typically involve caching specialists performing caching tasks, such as scrub jays (*Aphelocoma californica*), Eurasian jays (*Garrulus glandarius*) and black-capped chickadees (*Poecile atricapillus*) [27,29,30]. These results may be caused by specialized memory repertoires (cf. [23]). The second reason for rejecting the idea that non-human animals plan flexibly is that observed behaviour was not caused by human-like planning, but is best understood as results of associative learning, and that methodological shortcomings render these studies equivocal [31–34].

Why would an associative learning model be useful for understanding future oriented behaviour? Associative learning is well known for causing anticipatory behaviours, behaviours that can predict later meaningful events without immediate benefits [22,35]. Furthermore, self control, often mentioned as important for planning [28,36], can arise through associative learning [19]. It might be assumed that self-control is not possible through associative learning because immediately rewarded behaviour should always be preferred to non-rewarding behaviour. But, for many animals ‘wait’ or ‘stalk’ are behaviours that can be reinforced when followed by later possibilities for rewards. For example, predators learn stalking and waiting skills when they are young [37,38].

The model used here is an associative learning model capable of learning optimal behaviour in a complex world [19]. The model includes two different memories and a decision-making mechanism. One memory stores the associative strength of performing behaviour *B* towards stimulus *S*, and the other memory stores the estimated value of stimulus *S*. The model can learn behaviour sequences by linking single behaviours together through conditioned reinforcement (secondary reinforcement). This way, initially neutral stimuli that precede primary reinforcers can themselves become reinforcers, thereby modifying previously unrewarded behaviour [39–41]. For example, a clicker trained rabbit has heard clicks repeatedly prior to food rewards. For this rabbit, a click becomes rewarding in itself and the rabbit will learn to perform behaviours that only result in the rabbit hearing a click [42]. The model is further explained in the Material and methods section below.

Here I test the hypothesis that an associative learning model can account for results found in non-human planning studies. The learning model was used to simulate the outcomes of two planning studies, one with orangutans (*Pongo pygmaeus*) and bonobos (*Pan paniscus*) [24] and one with ravens (*Corvus corax*) [28]. The simulations were found to track key patterns within and between these studies. It is concluded that one cannot rule out that studies of flexible planning in apes and corvids can be accounted for by associative learning. Therefore, associative learning cannot only produce human-like behaviour (e.g. [1,2]) but is a candidate explanation for observations of planning and self-control in non-human animals.

## 2. Material and methods

Here I describe our learning model [19], the logic of the two different studies that were used for the simulations, and details of the simulations.

### 2.1. A description of the model

An animal has a behaviour repertoire and it can use its behaviours to navigate in a world of detectable environmental states. A behaviour takes the animal from one state to another. Each state, or stimuli, has a primary reinforcement value that is genetically fixed. These values can be negative, neutral or positive, and they guide learning so that behaviours favouring survival and reproduction are promoted. Animals are assumed to make choices that maximize the total value, and expectations of the value of a future state can develop [section 2.3. in 19]. The model can thus generate goal-directed behaviour (see [35, p. 32] for another discussion of goal-directed behaviour and learning).

In short, the model describes the learning of sequences of behaviour towards stimuli through changes in memory. It includes decision-making that takes memory into account to determine what behaviour should be selected when a given stimulus is perceived. Take for instance learning a single behaviour, such as when a dog learns to give its paw in response to the command ‘shake’. Lifting the paw is the behaviour, the command ’shake’ and the reward are stimuli. The event sequence to be learned is: command ‘shake’ → lift paw → reward, or

$$S_{\mathrm{command}\;‘\mathrm{shak}e^{\prime }}\to B_{\mathrm{lift}\;\mathrm{paw}}\to S_{\mathrm{food}\;\mathrm{reward}}$$

The model collects information about the value of performing behaviours towards different stimuli (or states), and information about the value of different stimuli (or being in specific states) [19]. Learning occurs through updates of two different kinds of memories. These memories correspond to Pavlovian and instrumental learning and are updated after an event sequence like in the dog example, or in general terms the event sequence *S* → *B* → *S*′. The first kind of memory is a stimulus–response association. We used *v*_*S*→*B*_ to denote the associative strength between stimulus *S* and behaviour *B*. In functional terms, *v*_*S*→*B*_ can be described as the estimated value of performing behaviour *B* when perceiving stimulus *S*. The second memory stores the value of a stimulus. We used *w*_*S*_ to denote this stimulus value and it is updated according to the value of a subsequent stimulus. In other words, *w*_*S*_ is the conditioned reinforcement value of being in state *S*. These memories are updated according to

2.1 $$\begin{array}{rl} \Delta v_{S\to B} & =\alpha_{v}(u_{S^{\prime }}+w_{S^{\prime }}-v_{S\to B})\\ \;\mathrm{and}\;\Delta w_{S} & =\alpha_{w}(u_{S^{\prime }}+w_{S^{\prime }}-w_{S}) \end{array}\}$$

after experiencing the event sequence *S* → *B* → *S*′. The stimulus–response association *v*_*S*→*B*_ is updated according to *u*_*S*′_ a primary inborn fixed value of stimulus *S*′, and *w*_*S*′_ the conditioned reinforcement value and the previously stored stimulus–response association *v*_*S*→*B*_. With conditioned reinforcement, the value of performing behaviour *B* when perceiving stimulus *S* is the sum of the primary and conditioned reinforcement value of stimulus *S*′. If only the first equation is used and *w* is excluded, then it represents instrumental stimulus–response learning, that is an instrumental version of the classic Rescorla–Wagner learning model [43,44]. The learning rates *α*_*v*_ and *α*_*w*_ determine the rate at which memory updates take place.

For the learning model to generate and select behaviour, a mechanism for decision-making is needed. We used a decision-making mechanism that selects behavioural responses and causes some variation in behaviour through exploration. This specifies the probability of behaviour *B* in state *S* as

2.2 $$\mathrm{Pr}(S\to B)=\frac{\exp(\beta v_{S\to B})}{\sum_{B^{\prime }}\exp(\beta v_{S\to B^{\prime }})},$$

which includes a parameter *β* that regulates the amount of exploration. All behaviours are equally likely to be selected if *β* = 0 without taking estimated values into account. If *β* is large, then the behaviour with the highest estimated value (*v*) will mainly be selected.

Let us return to the dog for a practical example. The dog hears the command ‘shake’, stimulus *S*. If the dog moves its paw upwards, that is performing behaviour *B*, it will receive the reward *S*′. The food reward *S*′ has a primary inborn value *u*. When the dog receives this reward after having responded correctly to the command ‘shake’, the stimulus–response memory *v*_command `shake′→lift paw_ will increase according to the top row in equation (2.1). In addition, the stimulus value *w* of the command ‘shake’ will be updated according to the bottom row of equation (2.1). This value *w* of command ’shake’ will approach the value *u* of the food reward, and thereby gain reinforcing properties in its own right; it has become a conditioned reinforcer. The conditioned reinforcer can pave the way for learning more behaviours before moving the paw upwards. This can happen because behaviours that result in the dog hearing the command ‘shake’ can be reinforced.

### 2.2. Simulating planning studies on great apes and ravens

The simulations of the planning experiments were based on detailed descriptions of the course of events in the two studies where key events were identified. Key events included what behaviours were trained before the tests and towards what objects, and what outcomes resulted from different choices during pretraining and tests. It is important to identify details in these studies [24,28], because test phases included a mix of rewarding and non-rewarding actions. Therefore, both stimulus–response (*v*) and stimulus values (*w*) were expected to change throughout the tests.

To both make the simulations possible and realistic, it was assumed that the animals entered these studies with some necessary everyday skills. It was assumed that the animals had, for example, previously learned to hold objects, how to move between rooms and compartments, where different things were located, and some basic skills regarding how to interact with the experimenters. The apes were for instance ushered out of the test room after choices to later be allowed back into the test room. By ignoring such everyday skills, the simulations and the behaviour descriptions were focused on the unique behaviour sequences the animals had to learn as part of the experiments.

The two studies [24,28] share key features. Before testing started, animals were subjected to pretraining. Here they learned to perform behaviours later scored as correct. Apart from the pretraining of correct behaviours, the raven study [28] also included extinction training. During extinction training, the ravens had the chance to learn that non-functional objects did not result in rewards. The key events in both studies used for scoring correct vs. incorrect choices were forced choice tests. Here the animals were forced to choose between one object they had previously learned could result in a reward, versus other objects that could not be used for later rewards (distractor objects). The ravens learned during extinction training that these distractor objects could not result in rewards. After the forced choice both studies included a time delay of some time, after which the animals were allowed to perform a behaviour using the previously chosen object. If an animal made a correct choice before the delay, it could later use its chosen object to get a reward. If an animal made an incorrect choice before the delay there were no opportunities for rewarding behaviours after the delay.

The simulations performed followed the pretraining phase and test phase of the studies. Comparisons are made with chance levels of correct choices set by the two studies. Mulcahy & Call [24] expected the apes to choose the correct by chance 25% of the times (one functional object and three distractor objects). Kabadayi & Osvath [28] expected the ravens to by chance make 25% correct choices in experiments 1 and 2, and 20% correct choices in experiment 3 and 4 (one functional object and three distractor objects in experiments 1 and 2, and 1 functional object, 1 small reward and three distractor objects in experiments 3 and 4). See simulation scripts for exact descriptions (see electronic supplementary material). To make it easier to follow the simulations here are in-depth descriptions of the two studies.

### 2.3. A description of Mulcahy and Call’s study on great apes

These tests were performed with orangutans and bonobos [24]. The study started with pretraining. Here an animal was placed in a test room and trained on two different tool tasks to get a reward from an apparatus. These functional tools will be referred to as functional objects. One task was to choose a tube and insert this tube into an apparatus. The other task was to choose a hook and use this to reach a bottle that could not be reached without having the hook. After pretraining, the animal was subjected to a forced choice test between functional objects and three corresponding non-functional objects (later referred to as distractor objects). But during this forced choice, access to the apparatus containing a reward was blocked. After the choice was made, the animal was ushered away from the test room into a waiting room. Objects not taken by the animal were now cleared from the test room. At this point, there was a delay. After the delay the animal was again allowed into the test room and given access to the apparatus. If a functional object had been chosen in the forced choice test, the animal could now use the object to get a reward, thereby exhibiting the behaviour it had learned during pretraining.

This study included four tests that were slightly different. Tests varied with respect to what tool was the functional object and the duration of delays. In addition, in the last test, the animals did not have to use the tool to get a reward. Note that here, in experiment 4, two new individuals were used and they did not take part in experiments 1, 2 or 3. This last part was of little importance here for reasons mentioned in the Results section. The simulations followed the logic of the study, and here are the details of the key events and delays used in the simulation:

Pretraining: Before tests, all subjects learned to use the functional tools. In two steps, a minimum of three plus eight pretraining trials were allowed for the tube task and a minimum of five pretraining trials were allowed for the hook task.
Experiment 1, tube condition: (1) Forced choice with functional tube and distractor objects (16 trials). (2) After choice go to another room. (3) Wait 1 h. (4) Return and if functional tube had been chosen this could be used to get a reward.
Experiment 2, tube condition: (1) Forced choice with functional tube and distractor objects (12 trials). (2) After choice go to another room. (3) Wait 14 h. (4) Return and if functional tube had been chosen this could be used to get a reward.
Experiment 3, hook condition: (1) Forced choice with functional hook and distractor objects (16 trials). (2) After choice go to another room. (3) Wait 1 h. (4) Return and if functional hook had been chosen this could be used to get a reward.
Experiment 4, hook condition: (1) Forced choice with functional hook and distractor objects (16 trials). (2) After choice go to another room. (3) Wait 1 h. (4) Return and if functional hook had been chosen a reward was received without using the hook.

The behaviour sequences to learn were the following:

Tube condition: *S*_tube_ → *B*_take tube_ → *S*_apparatus_ → *B*_use tube_ → *S*_reward_
Hook condition: *S*_hook_ → *B*_take hook_ → *S*_apparatus_ → *B*_use hook_ → *S*_reward_

In both conditions, the apes were never rewarded for choosing the distractor objects, or:

Distractors: *S*_distractor_ → *B*_take distractor_ → *S*_no reward_

### 2.4. A description of Kabadayi & Osvath’s study on ravens

These tests were performed with ravens [28]. This study started with pretraining. Here an animal was placed in a test room and trained on two different tool tasks to get a reward from an apparatus. As above, functional tools will be referred to as functional objects. One task was to put a stone in an apparatus to get a reward. The other task was to take a bottle cap (called token) and give it to a human. In contrast with the study on apes, before the tests started the ravens were also allowed extinction trials. Here an animal was allowed to interact with the objects that would be present during the forced choice tests, but that could never be used to get rewards (later referred to as distractor objects). After pretraining, the animal was subjected to a forced choice test between a functional object and three distractor objects. After a choice was made, the animal was not allowed to use the functional object for some time. In other words, no reward could be collected immediately after the choice test (with the exception of experiment 4). At this point, there was a delay. After the delay, the animal was allowed to use its chosen object. If a functional object had been chosen in the forced choice test, the animal could now use that object to get a reward, thereby exhibiting the behaviour it had learned during pretraining.

This study also included four tests that were slightly different. Tests varied with respect to the number of trials, the duration of delays, and in the last test, the animals did not have to wait before using a functional object to get a reward. It should be noted that in this study, two different rewards were used. One high value reward was used in pretraining and in all experiments. And in experiments 3 and 4, a known reward of little value was used in the forced choice situation alongside the functional tool and the distractor objects. Note that the experiments were not performed in the same order as they were numbered in the published study. I have chosen to present the tests in the temporal order in which they were performed (1,3,2,4). The simulations followed the logic of the study, and here are the details of the key events used in the simulation: the key events before and during the experiments were:

Pretraining: Before tests, all subjects learned to use the functional tools. In two steps, a minimum of three plus five pretraining trials were allowed for the tool task and 35 pretraining trials were allowed for the token task.
Extinction trials: In this phase, subjects were allowed to manipulate distractor objects for 5 min without receiving any rewards.
Experiment 1: (1) Forced choice with functional object and distractor objects. 14 trials in tool condition and 12 × 3 trials in token condition. (2) Wait 15 min. (3) Chosen object can be used again, and if the stone or token had been chosen it could be used to get a reward.
Experiment 3: (1) Forced choice with functional object, small reward and distractor objects. 14 trials in tool condition and 14 trials in token condition. (2) Wait 15 min. (3) Chosen object can be used again, and if the stone or token had been chosen it could be used to get a reward.
Experiment 2: (1) Forced choice with functional object and distractor objects. 6 trials in tool condition and 6 trials in token condition. (2) Wait 17 h. (3) Chosen object can be used again, and if the stone or token had been chosen it could be used to get a reward.
Experiment 4: (1) Forced choice with functional object, small reward, and distractor objects. 14 trials in tool condition and 14 trials in token condition. (2). If the stone or token had been chosen it could be used to get a reward.

The behaviour sequences to learn were the following:

Tool condition: *S*_tool_ → *B*_take tool_ → *S*_apparatus_ → *B*_use tool_ → *S*_reward_
Token condition: *S*_token_ → *B*_take token_ → *S*_human_ → *B*_give token_ → *S*_reward_

The ravens were also taught during an extinction phase that it was never rewarding choosing or using distractor objects. This was also the case during all tests, or:

Distractors: *S*_distractor_ → *B*_take distractor_ → *S*_no reward_

In the self-control phases of the study, the ravens had the opportunity to choose a small reward that was presented alongside the functional object (tool or token) and the distractor objects. Therefore, in experiments 3 and 4, these behaviour sequences were also possible:

Tool condition: *S*_dog kibble_ → *B*_take small reward_ → *S*_small reward_
Token condition: *S*_dog kibble_ → *B*_take small reward_ → *S*_small reward_

### 2.5. Illustration of memory updates during pretraining

To illustrate how these behaviour sequences are affected by learning, here is an example of memory updates for pretraining in the raven study. The behaviour sequence that developed during pretraining can be described as *S*_tool_ → *B*_take tool_ → *S*_apparatus_ → *B*_use tool_ → *S*_reward_ where the value of inserting the stone into the apparatus increased, so that $v_{S_{\mathrm{apparatus}}\;\to \;B_{\mathrm{use}\;\mathrm{tool}}}\gg 0$. As the model also includes conditioned reinforcement, the value of the stone itself is updated according to the value of the following stimulus, the large reward. With repeated experiences, the stimulus value (*w*) of *S*_reward_ will cause the stimulus value of *S*_tool_ to grow. As shown in our description of this model [19], with enough experiences the value of the tool will approximate the value of the large reward. By contrast, the extinction trials with repeated unrewarded experiences of the three distractor objects can be described as *S*_distractor_ → *B*_pick distractor_ → *S*_no reward_. This event sequence will cause a reduction in both the associative strength of choosing a distractor $v_{S_{\mathrm{distractor}}\;\to \;B_{\mathrm{pick}\;\mathrm{distractor}}}$ and the conditioned reinforcement value (*w*_distractor_) of the distractor. When the first test starts with a forced choice, the ravens’ behaviour was influenced by the pretraining with both the stone and the distractors.

### 2.6. Simulation details

The model above was incorporated in a Python program where learning occurred according to the detailed procedures of the two studies, as defined above, to get estimates of probabilities of choosing the different stimuli, and *v*- and *w*-values, throughout the studies. Two kinds of simulations were run. First simulations with the full model were run, and then simulations without stimulus values (*w*), that is only allowing our version of stimulus–response learning using only the first row in equation (2.1) together with decision-making (equation (2.2)). This was done to explore differences between our model that includes conditioned reinforcement [19] and a version of stimulus–response learning alone [43,44]. That version of stimulus–response learning is identical to the classic Rescorla–Wagner learning rule but in [19] we considered it in terms of an instrumental instead of a Pavlovian setting.

To account for delays, one time step per minute was included in the simulation at times of delay. During these time steps, only a background stimulus was experienced. This is not very important for the sake of memory updates because both stimulus–response and stimulus value memories are long-term memories. That animals remember stimulus–response associations and stimulus values for a very long time was not mentioned in either of the simulated studies [19].

The same learning parameters were used in all simulations. All behaviours started with an initial stimulus–response value *v* = 1, both *v*- and *w*-values were updated with learning rate *α* = 0.2, exploration was set to *β* = 1, and rewards were set to *u* = 6 apart from the low value rewards in experiments 3 and 4 in Kabadayi & Osvath [28] that were set to *u* = 2. Behaviour cost for all behaviours was 0.1 apart from passive responses that were set to 0 (see information for all behaviours and stimulus elements included in simulations in the electronic supplementary material). All simulations were run for 500 subjects and the number of trials followed approximately that of the experiments. That the number of trials did not perfectly match the empirical studies was due to the probabilistic nature of the decision-making equation. The lack of information of initial values of the animals makes exact quantitative comparisons difficult.

Although both the ravens and the apes had rich backgrounds, previously learned behaviour was ignored and initial values were assumed to be the same for distractor objects and functional objects. To be conservative, all associative strengths between behaviours and stimuli were assumed to be equal at the start of the simulations. Kabadayi & Osvath [28] did not calibrate the preferences of ravens with respect to the value of the two different food rewards, so there is no quantitative information about the differences between the rewards available. They stated in the method that the high quality food reward was both larger and more attractive. Exact information about the amount of extinction was lacking from the raven study, therefore it was assumed that the ravens had five extinction experiences with the distractors.

The behaviours and stimulus elements used in the simulations were as follows:

#### 2.6.1. Behaviours

Mulcahy & Call Tube: take tube, use tube, take distractor, being passive
Mulcahy & Call Hook: take hook, use hook, take distractor, being passive
Kabadayi & Osvath Tool: take tool, use tool, take distractor, being passive, take small reward
Kabadayi & Osvath Token: take token, use token, take distractor, being passive, take small reward

#### 2.6.2. Stimulus elements

Mulcahy & Call Tube: background, tube, tube task, distractor, reward
Mulcahy & Call Hook: background, hook, hook task, distractor, reward
Kabadayi & Osvath Tool: background, tool, apparatus, distractor, reward, small reward
Kabadayi & Osvath Token: background, token, human, distractor, reward, small reward

### 2.7. Data from the empirical studies

To compare the simulation results with the empirical data from the two studies [24,28], averages were calculated from the available data in the two respective studies (see figures in Results). This resulted in the average proportion of correct and incorrect choices in the forced choice tests. Note that experiment 4 in the ape study did not involve any correct behaviour using the tool upon returning to the apparatus after the delay, making this experiment difficult to interpret. In addition, data on choices for experiment 4 were not available in the text, therefore data from [24, fig. S2] was used for that data point. It is unfortunate to mix data this way but I chose this in favour of leaving data from experiment 4 out.

## 3. Results

**Figure 1.**
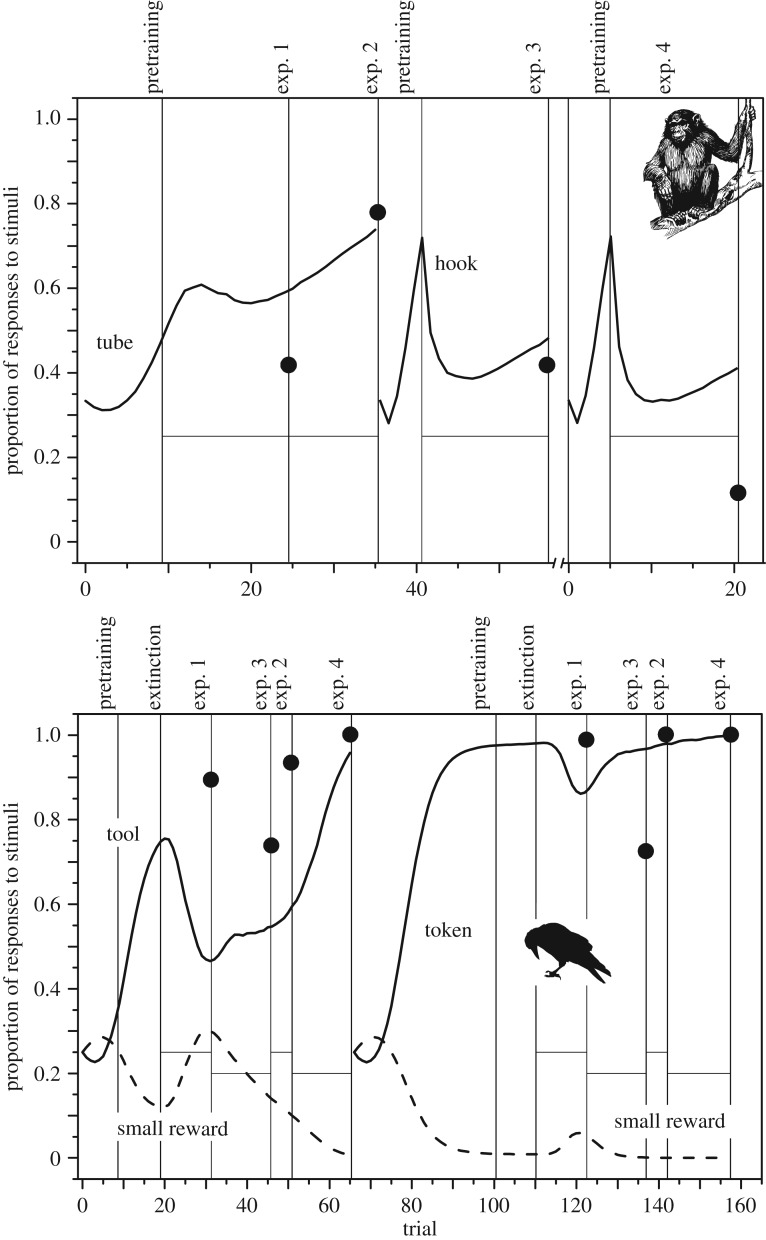
Results from empirical (dots) and simulation (lines) data showing the proportion of correct responses to functional objects, and for the raven study the simulated proportion of responses to small rewards (dashed lines). Bonobos and orangutans are in the top panel and ravens in the bottom panel. For the apes, choosing the tube was correct in experiments 1 and 2 (left line), and choosing a hook was the correct choice in experiments 3 and 4 (right line). Note that the *X*-axis of the top panel is broken, because experiment 4 was done with new individuals that only experienced pretraining prior to the experiment. For the ravens, the correct choice in the first half of the experiment was a tool (left line). A token was the correct choice in the second part of the experiment (right line). Horizontal lines are expected chance levels of correct choices during test phases (i.e. tube, hook, tool and token, respectively). Empirical data are averages of data from the end of each respective phase in the two studies [24,28]. Bonobo and raven graphics were downloaded from openclipart.org.

Overall, the simulations matched the results of both the raven and the great ape study. The simulations show how two factors together can contribute to the future directed behaviour exhibited by the great apes and ravens. First, conditioned reinforcement values of functional objects, established through pretraining and extinction, were capable of driving initial correct choices. This is shown in figure 1 where the proportion of correct choices is shown. Second, correct choices were rewarded throughout the studies, apart from experiment 4 in the ape experiment [24]. That the use of functional objects was rewarding throughout was sufficient for driving performance well above chance levels (figure 1). In the raven study, rewards delivered during the experiment account well for the near perfect performance in the two final parts of that study.

The fit was good between the empirical tests (shown as filled circles in figure 1) and simulations in that functional objects were more likely to be chosen than the distractor objects. The simulations also followed the general trends in that performance increased in the great ape study during experiments 1 and 2 and that performance was reduced in experiment 3. Although the simulations underestimated the performance in the tool condition of the raven study, the simulations followed closely the pattern in that performance was high in experiment 1, decreased in experiment 3 to reach nearly perfect performance in experiment 4. One reason for the simulation to have a lower success rate in the tool condition could be that the ravens were well trained and had rich backgrounds that are helpful in test situations. These birds were raised by humans and interact regularly with humans. They are also familiar with many different objects, experimental set-ups and rewards. By contrast, the simulations started assuming no previous knowledge. There was a close match between the simulations and the empirical data for the token condition, but the reduction in performance during experiment 3 was greater in the empirical data.

The simulations also captured that the great apes exhibited an overall lower success rate than the ravens did. At least two factors could have contributed to this difference. The apes experienced less pretraining than the ravens and, in contrast to the ravens, the apes were not allowed extinction training with the distractor objects prior to testing. This is shown in figure 1 where the probability of choosing the correct object is much higher at the start of experiment 1 in the raven study as compared with the ape study. That a lot of pretraining trials (35 in the token condition) combined with extinction trials can result in high performance in the forced choices is most clearly shown in the token condition of the raven study. Here the simulation tracked the observed high success rate closely.

**Figure 2.**
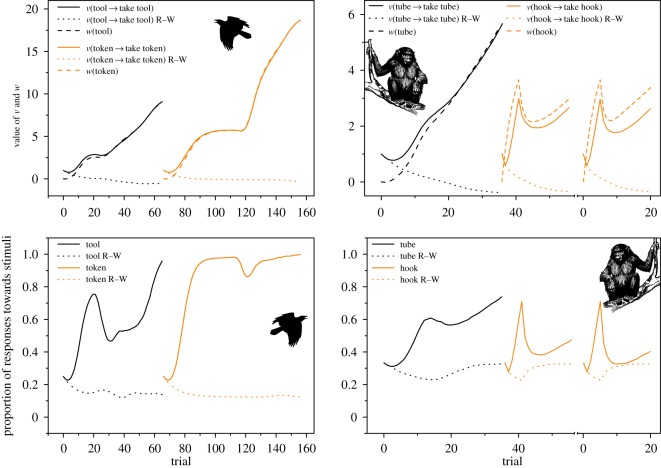
Results from the simulations to enable comparisons between the output from our learning model that includes conditioned reinforcement (stimulus values), with an instrumental version of the Rescorla–Wagner (R–W) model [19]. Simulations of the raven study are on the left and simulations of the ape study are on the right side. The top panels show memory updates: stimulus–response associations *v* for behaviours towards functional objects, and stimulus values *w* of these objects. As the functional objects are not themselves rewarding, simulations show that stimulus–response associations for choosing functional objects will not develop with the simpler learning model (R–W). And the bottom panels show that the stimulus–response learning model (R–W) cannot reproduce the behaviour patterns observed in the two studies, in stark contrast to our learning model that allows conditioned reinforcement. Experimental phases are the same as in figure 1, but here phases are not shown for clarity. Note that the *X*-axes in the right panels are broken because experiment 4 was done with new individuals that only experienced pretraining prior to the experiment. Raven and ape graphics were downloaded from openclipart.org.

Pretraining and extinction training did not only influence the likelihood of making correct decisions. Simulations reveal how pretraining and extinction also affect the proportion of choosing the incorrect objects, such as small rewards (figure 1). The effect of pretraining and extinction was most pronounced in the token condition of the raven study where the simulation suggests that the likelihood that the ravens should choose the small rewards over the functional objects was close to zero. The large amount of rewarding experiences with the functional objects (tool and token) resulted in large conditioned reinforcement values for these objects (figure 2). The simulations corroborated the pattern that ravens did not choose small rewards instead of functional objects, and that self-control is expected to emerge from associative learning.

The growth of stimulus–response values and stimulus values are shown in the top panel of figure 2.

Note that experiment 4 in the great ape study matches the simulations the least. Here two new apes were allowed to get the reward without using the previously functional tool and they returned with a correct tool 2 of 16 times, lower than in the simulation. This difference between empirical test and simulation could be reduced by increasing the cost of the behaviour. Increasing the cost of a behaviour that does not lead to a reward will lead to a reduction in performing the behaviour. But it is unclear what to expect from the animals in this situation when the apes face a situation with a less clear connection between a tool and a reward. And two of the four apes never attempted to solve the problem. To conclude, it is difficult to judge the precision and meaning of that data point (see [32, p. 922]).

The simulations also show the differences between associative learning models of different complexity. The limits of our version of stimulus–response learning [43,44] become obvious when compared with simulations using our learning model that incorporates both Pavlovian and instrumental learning [19]. In stimulus–response learning alone, behaviour sequences where a behaviour is not immediately followed by a reward cannot be learned (figure 2). For behaviour sequences to develop, stimuli more than one step before the reward need to become rewarding through conditioned reinforcement. When a previously neutral stimulus acquires a positive *w*-value, that is it becomes rewarding, it can drive the acquisition of positive *v*-values for behaviours that do not result in immediate rewards (top panel in figure 2). When comparing our model that can learn sequences of behaviour with the instrumental version of the Rescorla–Wagner model, it is clear that the probability of choosing the correct stimulus will not increase if only stimulus–response learning is allowed (figure 2). In addition, as *v*-values are only updated by the immediate reinforcer in stimulus–response learning, this also has the consequence that the small reward will be chosen in favour of the token and the tool, as the token and the tool cannot become valuable stimuli. This is shown in figure 2 as the incorrect choice of small rewards increases across trials when only our version of stimulus–response learning is allowed (marked with R–W in figure 2). Stimulus–response learning alone could not account for the results in neither the raven nor the ape study.

## 4. Discussion

Simulations of the two planning studies on ravens and great apes suggest that behaviour previously claimed to have been generated by flexible planning [24,28] can be accounted for by associative learning. As shown in artificial intelligence research and animal behaviour research, these models of associative learning are powerful in generating flexible behaviour sequences [1,19,45]. Therefore, the conclusion drawn in both the raven and great ape studies [24,28], that ravens and apes solve these problems by a specific flexible mechanism, has little support. Simulations performed here support critics that interpreted these results as consequences of associative learning [33,34]. If future studies aim at distinguishing associative processes from other kinds of mental mechanisms, they would benefit from improved experimental design including proper controls taking advantage of state-of-the-art learning models.

It was interesting to note that the simulations captured the difference between the study on ravens [28] and great apes [24]. This suggests that the simulations captured well the effects of pretraining-, extinction phases and rewards throughout the studies. High conditioned reinforcement values (*w*-values) for the correct objects (tool and token) and low values for the distractor objects were established before the first tests (figure 2). This was especially obvious in the token part of the raven experiment where the ravens were subjected to 35 pretraining trials where the behaviour sequence *S*_token_ → *B*_take token_ → *S*_human_ → *B*_give token_ → *S*_reward_ was consistently rewarded (lower panel, figure 1).

Another important factor for the positive results in the raven and great ape studies was that choosing the correct objects were rewarded throughout the tests. This maintained high *v*- and *w*-values for correct behaviours and correct objects, respectively. This also explains why the ravens neglected the small reward when presented together with the functional objects (figure 1). The functional objects led to rewards repeatedly throughout the study so they had acquired high stimulus values. As long as these values are higher than the value of the small reward, these functional objects will be chosen most of the time. However, with only stimulus–response learning—only allowing the updates of *v*-values as in the Rescorla–Wagner model—the small reward will be chosen because this model lacks conditioned reinforcement (figure 2). If one wants to avoid learning during tests, there are benefits with carrying out tests under extinction, as for instance in outcome revaluation studies (e.g. [46,47]). This way tests can reveal the consequences of prior experimental manipulations.

The results support the idea that self-control emerged through associative learning. We have previously shown how animals can, through associative learning, acquire self-control, given they are provided enough information and experiences [19, §2.3]. Kabadayi & Osvath [28] did not define self-control, but in a previous study [48] they defined it as ‘[ … ] the suppression of immediate drives in favour of delayed rewards’. This functional view of self-control fits many descriptions of behaviour in the animal behaviour literature. Observations of animals learning to reject small rewards when expecting large rewards, or in other words reject unprofitable prey when profitable prey are abundant, come from for instance fish (bluegill sunfish *Lepomis macrochirus*, [49]), crustaceans (shore crabs, *Carcinus maenas*, [50], and birds (great tits *Parus major*, [51] and redshanks *Tringa totanus*, [52]). These kinds of studies have to a large degree been ignored in studies where self-control is often studied as a separate kind of mental mechanism and not something that is subject to learning (e.g. [6,28,48]). Instead, in the light of these simulations, previous studies of self-control within animal cognition research (as e.g. [48]) may best be understood as being caused by learning including conditioned reinforcement [31].

Theoretically, self-control can develop in more than one way. Self-control can emerge through the acquisition of high conditioned reinforcement values for the functional objects. The functional object becomes more valuable than a small reward. But self-control can also emerge if for example ‘wait’ is considered as a behaviour in its own right. In this case, self-control can emerge through an increased *v*-value for ‘wait’ in the presence of a particular stimulus. Self-control in hunting cats might emerge through high *v*-values for waiting when subjected to a prey that is far away. More research is needed to better understand how different aspects of learning mechanisms interact to give rise to patterns of self-control. Genetic predispositions are likely to play a large role and interact with stimulus–response associations and stimulus values.

Another important result was that the difference between the ravens’ performance in experiment 3 and experiment 4 was captured by the simulations. The reason for the perfect performance in experiment 4 in both the raven study and the simulation was that the delay between choice and behaviour resulting in reward was omitted. Instead, there was an opportunity to use the object to collect a reward right after the forced choice. For this reason, every trial led potentially directly to rewards whereas choosing the correct object in experiment 3 was only rewarded after the delay. Or in other words, in experiments 1–3, the ravens could only get a reward every second time they chose the correct object, whereas in experiment 4 they got rewards every time and immediately after having chosen and used the functional item.

One similarity between our learning model and some reinforcement learning models in AI is that these mechanisms allow agents and animals to identify world states that are valuable, and what behaviours are productive in these valuable states. In an operational sense, these learning models generate planning when a behaviour (put in apparatus or give to human) towards a stimulus (stone or token) will produce high value food at a later stage. This happens despite the fact that the food (or another rewarding stimulus) is absent. Osvath & Kabadayi [53], in a reply to critics [33], defined flexible planning as ‘making decisions about futures outside one’s current sensory scope in domains for which one is not predisposed’. Irrespective of whether models come from AI [54] or animal behaviour [19], when conditioned reinforcement is included in learning models, planning behaviours that match this definition will emerge through the clever interplay of stimulus–response values and stimulus values. The key is that currently available stimuli can provide information about what behaviours should be performed to enter future valuable states. However, these learning models cannot simulate different outcomes mentally, they cannot travel mentally in time, nor reorganize information internally. To paraphrase Roberts [55], non-human animals can be ‘stuck in time’, while still exhibiting planning behaviour.

Mulcahy & Call [24] attempted to rule out instrumental conditioning as an explanation for the behaviour of the apes by performing experiment 4. This phase was similar to experiment 3, but the apes were not rewarded for using the functional tool. Instead of an ape entering the room with a functional tool that could be used to get a reward (as in experiment 3), an ape entered the room and found a reward if it had carried the functional tool to the test room from the waiting room. It was argued that if the apes performed better in the other experiments than in this one, it would suggest that the apes planned flexibly. Mulcahy & Call concluded their results ‘represent a genuine case of future planning’. A devil’s advocate could identify differences between experiments 3 and 4, rendering learning a more likely explanation. In experiment 3, the apes were explicitly rewarded for using the tool. This results in a high conditioned reinforcement value for the tool and a high stimulus–response value for using the tool on the apparatus. In experiment 4, however, Mulcahy & Call point out that there was a longer time between picking the tool up in the waiting room, carrying the tool to the test room, to subsequently get a reward without using the tool. Perhaps the low performance in experiment 4 was caused by the unclear connection between the tool and the reward, as the delay inhibits the acquisition of picking up the tool to later receive a reward. Proper control conditions are important to enable the rejection of hypotheses unambiguously (e.g. recent discussions in [56,57]). Our learning model can be used in future research to analyse such behavioural differences caused by variation in learning contingencies.

The simulations show that the ape study [24] and raven study [28] can be understood through associative learning. However, results from experiments with caching specialists [58,59], probably dependent upon genetic specializations [27,29,30], are currently beyond the scope of our learning model. Caching behaviour and feeding behaviour involve different motivational states in animals [60]. Motivational states can be regarded as internal stimuli and readily integrated in an associative learning model, which would result in increased flexibility in terms of making foraging and caching decisions. Our model does not include different motivational states in its current state, but we have given examples of how genetic predispositions can be integrated with the model [19, table 2]. One possible solution would be to introduce context-dependence, so that exploration is different for different external stimuli and/or for different internal states. Importantly, when making assumptions about more flexible mental mechanisms, the higher costs of exploration that are incurred by increased flexibility need to be taken into account (see [19, §3.3]). We expect that evolution has fine-tuned genetic predispositions that together with associative learning generate productive and species-specific behaviours.

Another important point for future studies is that when animals learn about consequences of behaviour, and stimulus–response values and stimulus values are updated, these are long-term memories (e.g. [61–63], see also [40]). A raven trained to give tokens to a human does not simply forget how to do this one day later. Behaviourally, the tool condition of the raven study is identical to when dog owners teach furry friends to ‘clean up’ by putting toys in a designated basket. Instead of the raven being rewarded for putting a stone in an apparatus, a dog gets a reward for putting a toy in a basket. Such long-term memories that are updated through associative learning are very different from the short-term memory of arbitrary stimuli [23].

In conclusion, the development of associative learning models is impressive in AI research and models have proven powerful in generating complex behaviour. One can ask why these powerful models are not more widely applied to non-human animal behaviour and why these models are underestimated as a cause of flexible behaviour in non-human animals. This is especially relevant given that research in animal cognition where non-human animals are claimed to have insights, exhibit causal reasoning, and the plan is criticized on a regular basis for suffering from grand claims based on a weak methodology (e.g. [31,64–70]). One way to solve this associative learning paradox is by integrating the fields of AI, animal learning, and animal cognition [71]. To understand mechanisms generating behaviour, formal bottom-up associative models are likely to be more illuminating than verbal top-down ‘higher-order’ cognitive models. For instance, because the latter models are more difficult to reject and they cannot be implemented in simulations or used when building robots. To sum up, it is concluded that one cannot rule out that flexible planning in apes and corvids, and probably many other species, emerges through associative learning.

## Supplementary Material

Supplementary materials for “What can associative learning do for planning?”

## Data accessibility

Figures and data from simulations can be generated using software and code as specified in the electronic supplementary material.

## References

[1] Mnih V *et al.* 2015 Human-level control through deep reinforcement learning. Nature 518, 529–533. (10.1038/nature14236) 25719670

[2] Silver D *et al.* 2016 Mastering the game of Go with deep neural networks and tree search. Nature 529, 484–489. (10.1038/nature16961) 26819042

[3] Silver D *et al.* 2017 Mastering chess and shogi by self-play with a general reinforcement learning algorithm. (http://arxiv.org/abs/1712.01815).

[4] Emery NJ, Clayton NS 2004 The mentality of crows: convergent evolution of intelligence in corvids and apes. Science 306, 1903–1907. (10.1126/science.1098410) 15591194

[5] Horner V, Carter JD, Suchak M, de Waal FB 2011 Spontaneous prosocial choice by chimpanzees. Proc. Natl Acad. Sci. USA 108, 13 847–13 851. (10.1073/pnas.1111088108) PMC3158226 21825175

[6] MacLean EL *et al.* 2014 The evolution of self-control. Proc. Natl Acad. Sci. USA 111, E2140–E2148. (10.1073/pnas.1323533111) 24753565 PMC4034204

[7] Suchak M, Eppley TM, Campbell MW, Feldman RA, Quarles LF, de Waal FB 2016 How chimpanzees cooperate in a competitive world. Proc. Natl Acad. Sci. USA 113, 10 215–10 220. (10.1073/pnas.1611826113) 27551075 PMC5018789

[8] Whiten A 2017 Social learning and culture in child and chimpanzee. Annu. Rev. Psychol. 68, 129–154. (10.1146/annurev-psych-010416-044108) 28051932

[9] Allen C, Bekoff M 1995 Cognitive ethology and the intentionality of animal behaviour. Mind Lang. 10, 313–328. (10.1111/j.1468-0017.1995.tb00017.x)

[10] Tomasello M, Call J 1997 Primate cognition. Oxford, UK: Oxford University Press.

[11] Mulcahy NJ, Call J 2006 How great apes perform on a modified trap-tube task. Anim. Cogn. 9, 193–199. (10.1007/s10071-006-0019-6) 16612632

[12] Bird CD, Emery NJ 2009 Insightful problem solving and creative tool modification by captive nontool-using rooks. Proc. Natl Acad. Sci. USA 106, 10 370–10 375. (10.1073/pnas.0901008106) 19478068 PMC2700937

[13] Bird CD, Emery NJ 2009 Reply to Lind et al.: insight and learning. Proc. Natl Acad. Sci. USA 106, E77–E77. (10.1073/pnas.0906351106) PMC2710624 19571011

[14] Jelbert SA, Taylor AH, Cheke LG, Clayton NS, Gray RD 2014 Using the Aesop’s fable paradigm to investigate causal understanding of water displacement by New Caledonian crows. PLoS ONE 9, e92895 (10.1371/journal.pone.0092895) 24671252 PMC3966847

[15] Heyes C 2012 Simple minds: a qualified defence of associative learning. Phil. Trans. R. Soc. B 367, 2695–2703. (10.1098/rstb.2012.0217) 22927568 PMC3427553

[16] Heyes C 2012 What’s social about social learning? J. Comp. Psychol. 126, 193–202. (10.1037/a0025180) 21895355

[17] Ghirlanda S, Enquist M, Lind J 2013 Coevolution of intelligence, behavioral repertoire, and lifespan. Theor. Popul. Biol. 91, 44–49. (10.1016/j.tpb.2013.09.005) 24044983

[18] Koops K, Furuichi T, Hashimoto C 2015 Chimpanzees and bonobos differ in intrinsic motivation for tool use. Sci. Rep. 5, 11356 (10.1038/srep11356) 26079292 PMC4468814

[19] Enquist M, Lind J, Ghirlanda S 2016 The power of associative learning and the ontogeny of optimal behaviour. R. Soc. open sci. 3, 160734 (10.1098/rsos.160734) 28018662 PMC5180160

[20] McCormack T, Hoerl C, Butterfill S 2011 Tool use and causal cognition. Oxford, UK: Oxford University Press.

[21] Carew TJ, Sahley CL 1986 Invertebrate learning and memory: from behavior to molecules. Annu. Rev. Neurosci. 9, 435–487. (10.1146/annurev.neuro.9.1.435) 2423010

[22] Bouton ME 2007 Learning and behavior: a modern synthesis. Sinauer, MA: Sunderland.

[23] Lind J, Enquist M, Ghirlanda S 2015 Animal memory: a review of delayed matching-to-sample data. Behav. Processes 117, 52–58. (10.1016/j.beproc.2014.11.019) 25498598

[24] Mulcahy NJ, Call J 2006 Apes save tools for future use. Science 312, 1038–1040. (10.1126/science.1125456) 16709782

[25] Naqshbandi M, Roberts WA 2006 Anticipation of future events in squirrel monkeys (*Saimiri sciureus*) and rats (*Rattus norvegicus*): tests of the Bischof-Köhler hypothesis. J. Comp. Psychol. 120, 345–357. (10.1037/0735-7036.120.4.34) 17115855

[26] Raby CR, Alexis DM, Dickinson A, Clayton NS 2007 Planning for the future by western scrub-jays. Nature 445, 919–921. (10.1038/nature05575) 17314979

[27] Bourjade M, Call J, Pelé M, Maumy M, Dufour V 2014 Bonobos and orangutans, but not chimpanzees, flexibly plan for the future in a token-exchange task. Anim. Cogn. 17, 1329–1340. (10.1007/s10071-014-0768-6) 24942106

[28] Kabadayi C, Osvath M 2017 Ravens parallel great apes in flexible planning for tool-use and bartering. Science 357, 202–204. (10.1126/science.aam8138) 28706072

[29] Premack D 2007 Human and animal cognition: continuity and discontinuity. Proc. Natl Acad. Sci. USA 104, 13 861–13 867. (10.1073/pnas.0706147104) PMC1955772 17717081

[30] Suddendorf T, Corballis MC 2010 Behavioural evidence for mental time travel in nonhuman animals. Behav. Brain Res. 215, 292–298. (10.1016/j.bbr.2009.11.044) 19962409

[31] Suddendorf T, Corballis MC, Collier-Baker E 2009 How great is great ape foresight? Anim. Cogn. 12, 751–754. (10.1007/s10071-009-0253-9) 19565281

[32] Cheke LG, Clayton NS 2010 Mental time travel in animals. Wiley Interdiscip. Rev. Cogn. Sci. 1, 915–930. (10.1002/wcs.59) 26271786

[33] Redshaw J, Taylor AH, Suddendorf T 2017 Flexible planning in ravens? Trends Cogn. Sci. 21, 821–822. (10.1016/j.tics.2017.09.001) 28927634

[34] Suddendorf T, Bulley A, Miloyan B 2018 Prospection and natural selection. Curr. Opin. Behav. Sci. 24, 26–31. (10.1016/j.cobeha.2018.01.019)

[35] Pearce JM 2008 Animal learning and cognition, 3rd edn Hove, UK: Psychology Press.

[36] Shettleworth S 2010 Cognition, evolution, and behavior. Oxford, UK: Oxford University Press.

[37] Fox M 1969 Ontogeny of prey-killing behavior in Canidae. Behaviour 35, 259–272. (10.1163/156853969X00233)

[38] Eaton RL 1970 The predatory sequence, with emphasis on killing behavior and its ontogeny, in the cheetah (*Acinonyx jubatus* Schreber). Zeitschrift für Tierpsychologie 27, 492–504. (10.1111/j.1439-0310.1970.tb01883.x)

[39] Kelleher RT, Gollub LR 1962 A review of positive conditioned reinforcement. J. Exp. Anal. Behav. 5, 543–597. (10.1901/jeab.1962.5-s543) 14031747 PMC1404082

[40] Mackintosh NJ 1974 The psychology of animal learning. London, UK: Academic Press.

[41] Williams BA 1994 Conditioned reinforcement: experimental and theoretical issues. Behav. Anal. 2, 261–285. (10.1007/bf03392675) PMC2733461 22478192

[42] McGreevy P, Boakes R 2011 Carrots and sticks: principles of animal training. Sydney, Australia: Darlington Press.

[43] Rescorla RA, Wagner AR 1972 A theory of Pavlovian conditioning: variations in the effectiveness of reinforcement and nonreinforcement. In *Classical conditioning II: current research and theory* (eds AH Black, WF Prokasy), pp. 64–99. New York, NY: Appleton-Century-Crofts.

[44] Blough DS 1975 Steady state data and a quantitative model of operant generalization and discrimination. J. Exp. Psychol. Anim. Behav. Process. 104, 3–21. (10.1037/0097-7403.1.1.3)

[45] Sutton RS, Barto AG 1998 Reinforcement learning. Cambridge, MA: MIT Press.

[46] Balleine B, Dickinson A 1991 Instrumental performance following reinforcer devaluation depends upon incentive learning. Q. J. Exp. Psychol. 43, 279–296. (10.1080/14640749108401271)

[47] Dickinson A, Balleine B 1994 Motivational control of goal-directed action. Anim. Learn. Behav. 22, 1–18. (10.3758/BF03199951)

[48] Osvath M, Osvath H 2008 Chimpanzee (*Pan troglodytes*) and orangutan (*Pongo abelii*) forethought: self-control and pre-experience in the face of future tool use. Anim. Cogn. 11, 661–674. (10.1007/s10071-008-0157-0) 18553113

[49] Werner EE, Hall DJ 1974 Optimal foraging and the size selection of prey by the bluegill sunfish (*Lepomis macrochirus*). Ecology 55, 1042–1052. (10.2307/1940354)

[50] Elner RW, Hughes RN 1978 Energy maximization in the diet of the shore crab *Carcinus maenas*. J. Anim. Ecol. 47, 103–116. (10.2307/3925)

[51] Krebs JR, Erichsen JT, Webber MI, Charnov EL 1977 Optimal prey selection in the great tit (*Parus major*). Anim. Behav. 25, 30–38. (10.1016/0003-3472(77)90064-1)

[52] Goss-Custard JD 1977 Optimal foraging and the size selection of worms by redshank, *Tringa totanus*, in the field. Anim. Behav. 25, 10–29. (10.1016/0003-3472(77)90063-x)

[53] Osvath M, Kabadayi C 2018 Contrary to the gospel, ravens do plan flexibly. Trends Cogn. Sci. 22, 474–475. (10.1016/j.tics.2018.03.011) 29680766

[54] Barto AJ 2003 Reinforcement learning. In *The handbook of brain theory and neural networks* (ed. MA Arbib), pp. 963–968. Cambridge, MA: MIT Press.

[55] Roberts WA 2002 Are animals stuck in time? Psychol. Bull. 128, 473–489. (10.1037/0033-2909.128.3.473) 12002698

[56] Ghirlanda S, Lind J 2017 ‘Aesop’s fable’ experiments demonstrate trial-and-error learning in birds, but no causal understanding. Anim. Behav. 123, 239–247. (10.1016/j.anbehav.2016.10.029)

[57] Hennefield L, Hwang HG, Weston SJ, Povinelli DJ 2018 Meta-analytic techniques reveal that corvid causal reasoning in the Aesop’s fable paradigm is driven by trial-and-error learning. Anim. Cogn. 21, 735–748. (10.1007/s10071-018-1206-y) 30132156 PMC6181768

[58] Correia SP, Dickinson A, Clayton NS 2007 Western scrub-jays anticipate future needs independently of their current motivational state. Current Biology 17, 856–861. (10.1016/j.cub.2007.03.063) 17462894

[59] Cheke LG, Clayton NS 2012 Eurasian jays (*Garrulus glandarius*) overcome their current desires to anticipate two distinct future needs and plan for them appropriately. Biol. Lett. 8, 171–175. (10.1098/rsbl.2011.0909) 22048890 PMC3297405

[60] Clayton NS, Dickinson A 1999 Motivational control of caching behaviour in the scrub jay *Aphelocoma coerulescens*. Anim. Behav. 57, 435–444. (10.1006/anbe.1998.0989) 10049484

[61] Skinner BF 1950 Are theories of learning necessary? Psychol. Rev. 57, 193–216. 15440996 10.1037/h0054367

[62] Vaughan W, Greene SL 1984 Pigeon visual memory capacity. J. Exp. Psychol. Anim. Behav. Process. 10, 256–271. (10.1037/0097-7403.10.2.256)

[63] Gleitman H 1971 Forgetting of long-term memories in animals. In *Animal memory* (eds W Honig, P James), pp. 1–44. New York, NY: Academic Press.

[64] Penn DC, Holyoak KJ, Povinelli DJ 2008 Darwin’s mistake: explaining the discontinuity between human and nonhuman minds. Behav. Brain Sci. 31, 109–130. 18479531 10.1017/S0140525X08003543

[65] Wynne C 2008 Aping Language: a skeptical analysis of the evidence for nonhuman primate language. Skeptic 13, 10–15.

[66] Lind J, Ghirlanda S, Enquist M 2009 Insight learning or shaping? Proc. Natl Acad. Sci. USA 106, E76 (10.1073/pnas.0906120106) 19571011 PMC2710624

[67] Shettleworth SJ 2010 Clever animals and killjoy explanations in comparative psychology. Trends Cogn. Sci. 14, 477–481. (10.1016/j.tics.2010.07.002) 20685155

[68] Manger P 2013 Questioning the interpretations of behavioral observations of cetaceans: is there really support for a special intellectual status for this mammalian order? Neuroscience 250, 664–696. (10.1016/j.neuroscience.2013.07.041) 23896571

[69] Dymond S, Stewart I 2016 Relational and analogical reasoning in comparative cognition. Int. J. Comp. Psychol. 29, 1–11.

[70] Lindenfors P 2017 Bird brains: are crows as intelligent as some scientists claim? Skept. Mag. 22, 10–11.

[71] Lind J, Enquist M 2009 More synthetic work is needed. Adapt. Behav. 17, 329–330. (10.1177/1059712309340860)

