# Supplementary material for: What can associative learning do for planning?
Source: R Soc Open Sci. 2018 Nov 28;5(11):180778. doi: 10.1098/rsos.180778 (PMC6281940; doi:10.1098/rsos.180778)
Supplement: Supplementary materials for “What can associative learning do for planning?” [file rsos180778supp1.pdf]

Supplementary materials for “What can associative learning do for planning?”

Johan Lind

October 23, 2018

Contents

1 Introduction **1**

2 Learning simulator **1**

3 Simulations **2**

3.1 Mulcahy & Call 2006: Experiment 1 and 2 2

3.2 Mulcahy & Call 2006: Experiment 3 and 4 4

3.3 Kabadayi & Osvath 2017: The tool condition 6

3.4 Kabadayi & Osvath 2017: Simulating the token condition 8

1 Introduction

This document presents the program and contains the code used for simulations in the paper “What can associative learning do for planning?”. Together with the learning simulator program, these scripts produce figures[1]. All simulations are based on the mechanisms described in Enquist M, Lind J & Ghirlanda S 2016 ’The power of associative learning and the ontogeny of optimal behaviour.’ *Royal Society Open Science*, 3(11), 160734.

The two studies that were simulated using the scripts below are: Mulcahy & Call 2006 ’Apes save tools for future use.’ Science 312, 1038-1040 and Kabadayi & Osvath 2017 ’Ravens parallel great apes in flexible planning for tool-use and bartering.’ Science 357, 202-204.

2 Learning simulator

The scripts are to be used in a computer program made to simulate learning phenomena. The simulations are based on defining a world, and parameters for

[1] Output data files can also be produced by using export commands. See details in user guide for the learning simulator.

the organism that is subjected to that world, following the formalism described in the paper. The world is defined according to what stimuli are present, and what the consequences are when subjected to these stimuli. The organism is defined according to what behaviors it can perform, what its initial conditions are, and parameters that determine memory updates and exploration. Different learning mechanisms can be used. In this paper I used stimulus-response learning and combined stimulus-response- and stimulus value learning (combining instrumental and Pavlovian conditioning).

The simulator can produce both figures and data. Please note that the figures produced by the scripts are similar, but not identical to the ones in the paper.

The learning simulator can be downloaded at:

`https://github.com/markusrobertjonsson/learning_simulator`.

Running the learning simulator requires Python 3.5 (`https://www.python.org/downloads/`) and Matplotlib (`https://matplotlib.org/`). With Python and Matplotlib installed, the file `lesim.py` opens the learning simulator where the scripts below can be used. For details about the learning simulator and how to run the scripts see the user guide 'ug.pdf' that is included in the learning simulator package.

3 Simulations

All learning parameters used in the simulations are specified in each script. Simulations were done using two different learning mechanisms included in the learning simulator. Simulations were either performed using the instrumental version of the Rescorla-Wagner model, that is including only updates of stimulus-response associations ('SR' in the scripts) or using both updates of stimulus-response associations and stimulus values ('ga'[2] in the scripts).

The scripts are presented in the order in which they appear in the manuscript. Each script is presented briefly. For rationale and full descriptions of the simulations see text in the paper "What can associative learning do for planning?". Please note line breaking of long lines in the scripts below. Hard line breaks must be fixed for the scripts to run in the learning simulator.

3.1 Mulcahy & Call 2006: Experiment 1 and 2

```
@parameters
{
'subjects'          : 500, # number of individuals
'mechanism'         : 'ga',
'behaviors'         : ['taketube','usetube','takedist','0'],
'stimulus_elements' : ['newtrial','background','tube','tubetask','
    distractor','reward'],
'start_v'           : {'default':1},
```

[2] 'ga' is short for genetically guided associative learning.

```
'alpha_v'             :  0.2,
'alpha_w'             :  0.2,
'beta'                :  1,
'behavior_cost'       :  {'default':0,'taketube':0.1,'usetube':0.1,'
    takedist':0.1},
'u'                   :  {'reward':6, 'default': 0},
'omit_learning'       :  ['newtrial'],
'response_requirements':{'taketube':['tube'],'takedist':['
    distractor'],'usetube':['tubetask']} }

# Pretraining with tubetask, assumed to be 3 trials with just "
    usetube"
# S(tubetask)->B(usetube)->S(reward)
#@phase {'label':'pretrainingtube1', 'end':'reward=3'}
#NEW_TRIAL      newtrial                  | START
#START          tube                      | TUBETASK                 |
    END
#TUBETASK       tubetask                  | 'usetube': PRETRAINING1 |
    END
#PRETRAINING1   reward                    | END
#END            background                | NEW_TRIAL

# Pretraining with tube and tubetask, 8 trials with behavior
    sequence
# S(tube)->B(taketube)->S(tubetask)->B(usetube)->S(reward)
@phase {'label':'pretrainingtube2', 'end':'reward=8'}
NEW_TRIAL      newtrial                  | START
START          tube                      | 'taketube': TUBETASK
    |END
TUBETASK       tubetask                  | 'usetube': PRETRAINING2
    |END
PRETRAINING2   reward                    | END
END            background                | NEW_TRIAL

# Experiment 1, 16 trials with tube and distractors
@phase {'label':'experiment1', 'end':'newtrial=16'}
NEW_TRIAL      newtrial                  | START
START          ('tube','distractor')     | 'taketube':WAIT
    |END
WAIT           background                | 60:TUBE
    |WAIT
TUBE           tube                      | 'taketube':TUBETASK
    |END
TUBETASK       tubetask                  | 'usetube':REWARD
    |END
REWARD         reward                    | END
END             background                | NEW_TRIAL

# Experiment 2, 12 trials with tube and distractors
@phase {'label':'experiment2', 'end':'newtrial=12'}
NEW_TRIAL      newtrial                  | START
START          ('tube','distractor')     | 'taketube': WAIT
    |END
WAIT           background                | 840:TUBE
    |WAIT
TUBE           tube                      | 'taketube':TUBETASK
    |END
```

```
TUBETASK        tubetask                    | 'usetube':REWARD
    |END
REWARD          reward                      | END
END             background                  | NEW_TRIAL

@run

@figure 'v values'
@vplot ('tube','taketube') {'steps':'newtrial'}
@vplot ('tube','0') {'steps':'newtrial'}
@vplot ('tubetask','0') {'steps':'newtrial'}
@vplot ('tubetask','usetube') {'steps':'newtrial'}
@vplot ('distractor','takedist') {'steps':'newtrial'}
@legend #('taketube','takedist','0','0')

@figure 'Prob. responding'
@pplot (('tube','distractor'),'taketube') {'steps':'newtrial'}
@pplot (('tube','distractor'),'takedist') {'steps':'newtrial'}
@legend #('tube', 'distractor')

@figure 'w values'
@wplot ('tube') {'steps':'newtrial'}
@wplot ('distractor') {'steps':'newtrial'}
@wplot ('tubetask')  {'steps':'newtrial'}
@legend #('tube','distractor','tubetask')
```

3.2 Mulcahy & Call 2006: Experiment 3 and 4

Note that there were new test subjects for experiment 4. This is the reason for keeping simulations of experiment 3 and 4 separate from each other.

```
@parameters
{
'subjects'           : 500, # number of individuals
'mechanism'          : 'ga',
'behaviors'          : ['takehook','usehook','takedist','0'],
'stimulus_elements'  : ['newtrial','background','hook','hooktask','
    distractor','reward'],
'start_v'            : {'default':1},
'alpha_v'            : 0.2,
'alpha_w'            : 0.2,
'beta'               : 1,
'behavior_cost'      : {'default':0,'takehook':0.1,'usehook':0.1,'
    takedist':0.1},
'u'                  : {'reward':6, 'default': 0},
'omit_learning'      : ['newtrial'],
'response_requirements':{'takehook':['hook'],'takedist':['
    distractor'],'usehook':['hooktask']}
}

# *Pretraining with hook and hooktask. 5 trials with behavior
    sequence
@phase {'label':'pretraininghook', 'end':'reward=5'}
NEW_TRIAL     newtrial                      | START
START         hook                          | 'takehook': HOOKTASK
    |START
```

```
HOOKTASK       ('hook','hooktask')          | 'usehook': PRETRAINING
     |HOOKTASK
PRETRAINING   reward                        | END
END           background                    | NEW_TRIAL

# Experiment 3, 16 trials with hook and distractors
@phase {'label':'experiment3', 'end':'newtrial=16'}
NEW_TRIAL      newtrial                     | START
START          ('hook','distractor')        | 'takehook':WAIT
     |END
WAIT           background                   | 60:HOOK
     |WAIT
HOOK           hook                         | 'takehook':HOOKTASK
     |END
HOOKTASK       hooktask                     | 'usehook':REWARD
     |END
REWARD         reward                       | END
END             background                  | NEW_TRIAL

# Experiment 4, 16 trials with tube and distractors
@phase {'label':'experiment4', 'end':'newtrial=16'}
NEW_TRIAL      newtrial                     | START
START          ('hook','distractor')        | 'takehook': WAIT
     |END
WAIT           background                   | 60:HOOK
     |WAIT
HOOK           hook                         | 'takehook':BACKGROUND
     |END
BACKGROUND     background                   | '0':REWARD
     |END
REWARD         reward                       | END
END             background                  | NEW_TRIAL

@run  {'phases':('pretraininghook','experiment3'), 'label':'Exp3'}
@run  {'phases':('pretraininghook','experiment4'), 'label':'Exp4'}

@figure 'Prob. responding'
@pplot (('hook','distractor'),'takehook') {'runlabel':'Exp3','steps
     ':'newtrial'}
@pplot (('hook','distractor'),'takehook') {'runlabel':'Exp4','steps
     ':'newtrial'}
@legend ('Exp 3', 'Exp 4')

@figure 'v values' @vplot ('hook','takehook') {'runlabel':'Exp3','
     steps':'newtrial'}
@vplot ('hook','takehook') {'runlabel':'Exp4','steps':'newtrial'}
#@vplot ('hooktask','usehook') {'steps':'newtrial'}
#@vplot ('distractor','takedist') {'steps':'newtrial'}
#@vplot ('hook','0') {'steps':'newtrial'}
#@vplot ('hooktask','0') {'steps':'newtrial'}
@legend ('takehook Exp3','takehook Exp4')

@figure 'w values'
@wplot ('hook') {'runlabel':'Exp3','steps':'newtrial'}
@wplot ('hook') {'runlabel':'Exp4','steps':'newtrial'}
#@wplot ('distractor') {'steps':'newtrial'}
#@wplot ('hooktask')  {'steps':'newtrial'}
```

```
#@wplot ('reward')  {'steps':'newtrial'}
@legend ('hook exp 3','hook exp 4')
```

3.3 Kabadayi & Osvath 2017: The tool condition

```
@parameters
{
'subjects'          : 500, # number of individuals
'mechanism'         : 'ga',
'behaviors'         : ['taketool','usetool','takedist','takesmall
    ','0'],
'stimulus_elements' : ['newtrial','background','tool','apparatus','
    distractor','reward','smallreward'],
'start_v'           : {'default':1},
'alpha_v'           : 0.2,
'alpha_w'           : 0.2,
'beta'              : 1,
'behavior_cost'     : {'default':0,'taketool':0.1,'takedist':0.1,'
    takesmall':0.1,'usetool':0.1},
'u'                 : {'reward':6, 'smallreward':2, 'default': 0},
'omit_learning'     : ['newtrial'],
'response_requirements':{'taketool':['tool'],'takedist':['
    distractor'],'takesmall':['smallreward'],'usetool':['apparatus
    ']}
}

# Pretraining with tool, with a minimum of 8 (minumum of 3+5)
    trials with high quality reward
@phase {'label':'pretrainingtool', 'end':'reward=8'}
NEW_TRIAL      newtrial                   | START
START          tool                       | 'taketool': APPARATUS
    |END
APPARATUS      apparatus                  | 'usetool': PRETRAINING1
    |END
PRETRAINING1   reward                     | END
END            background                 | NEW_TRIAL

# Extinction in tool condition, 10 trials with distractor no reward
@phase {'label':'pretrainingdistractor', 'end':'newtrial=10'}
NEW_TRIAL      newtrial                   | START
START          distractor                 | 'takedist': PRETRAINING1
    |END
PRETRAINING1   background                 | END
END            background                 | NEW_TRIAL

# Experiment 1, 14 trials with tool and apparatus, delay=15 minutes
@phase {'label':'experiment1', 'end':'newtrial=14'}
NEW_TRIAL      newtrial                   | START
START          ('tool','distractor')      | 'taketool': WAIT
    |END
WAIT           background                 | 15:TOOL
    |WAIT
TOOL           tool                       | 'taketool':APPARATUS
    |END
APPARATUS      apparatus                  | 'usetool':REWARD
    |END
```

```
REWARD          reward                          | END
END              background                     | NEW_TRIAL

# Experiment 3, 14 trials with tool and apparatus, delay=15 minutes
@phase {'label':'experiment3', 'end':'newtrial=14'}
NEW_TRIAL       newtrial                                        | START
START           ('tool','distractor','smallreward')             | 'taketool':
     WAIT         |'takesmall':SMALLREWARD |END
SMALLREWARD     smallreward                                     | END
WAIT            background                                      | 15:TOOL
               |WAIT
TOOL            tool                                            | 'taketool':
    APPARATUS     |END
APPARATUS       apparatus                                       | 'usetool':
    REWARD          |END
REWARD          reward                                          | END
END              background                                      | NEW_TRIAL

# Experiment 2, 6 trials with tool and apparatus, delay=17 hours
@phase {'label':'experiment2', 'end':'newtrial=6'}
NEW_TRIAL       newtrial                        | START
START           ('tool','distractor')           | 'taketool': WAIT
     |END
WAIT            background                      | 1020:TOOL
     |WAIT
TOOL            tool                            | 'taketool':APPARATUS
     |END
APPARATUS       apparatus                       | 'usetool':REWARD
     |END
REWARD          reward                          | END
END              background                     | NEW_TRIAL

# Experiment 4, 14 trials with tool, small reward and distractors,
    no delay between taketoken and reward
@phase {'label':'experiment4', 'end':'newtrial=14'}
NEW_TRIAL       newtrial                                        | START
START           ('tool','distractor','smallreward')             | 'taketool':
    APPARATUS     |'takesmall':SMALLREWARD |END
APPARATUS       apparatus                                       | 'usetool':
    REWARD      |END
SMALLREWARD     smallreward                                     | END
REWARD          reward                                          | END
END              background                                      | NEW_TRIAL

@run

@figure 'v values'
@vplot ('tool','taketool') {'steps':'newtrial'}
@vplot ('apparatus','usetool') {'steps':'newtrial'}
@vplot ('distractor','takedist') {'steps':'newtrial'}
@vplot ('smallreward','takesmall') {'steps':'newtrial'}
@legend #('taketool','takedist','takesmall') {'steps':'newtrial'}

@figure 'Prob. responding'
@pplot (('tool','distractor','smallreward'),'taketool') {'steps':'
    newtrial'}
@pplot (('tool','distractor','smallreward'),'takedist') {'steps':'
```

```
    newtrial'}
@pplot (('tool','distractor','smallreward'),'takesmall') {'steps':'
    newtrial'}
@legend #('tool', 'background','distractor')

@figure 'w values'
@wplot ('tool') {'steps':'newtrial'}
@wplot ('apparatus') {'steps':'newtrial'}
@wplot ('distractor') {'steps':'newtrial'}
@legend #('tool','apparatus')
```

3.4 Kabadayi & Osvath 2017: Simulating the token condition

```
@parameters
{
'subjects'              : 500, # number of individuals
'mechanism'             : 'ga',
'behaviors'             : ['taketoken','givetoken','takedist','
    takesmall','0'],
'stimulus_elements'     : ['newtrial','background','token','human','
    distractor','reward','smallreward'],
'start_v'               : {'default':1},
'alpha_v'               : 0.2,
'alpha_w'               : 0.2,
'beta'                  : 1,
'behavior_cost'         : {'default':0,'taketoken':0.1,'takedist':0.1,'
    takesmall':0.1,'givetoken':0.1},
'u'                     : {'reward':6, 'smallreward':2, 'default': 0},
'omit_learning'         : ['newtrial'],
'response_requirements':{'taketoken':['token'],'takedist':['
    distractor'],'takesmall':['smallreward'],'givetoken':['human']}
}

# Pretraining with token, 35 trials with high quality reward
@phase {'label':'pretrainingtool', 'end':'reward=35'}
NEW_TRIAL       newtrial                    | START
START           token                       | 'taketoken': HUMAN
            |END
HUMAN           human                       | 'givetoken':
    PRETRAINING1 |END
PRETRAINING1    reward                      | END
END             background                  | NEW_TRIAL

# Extinction in tool condition, 10 trials with distractor no reward
@phase {'label':'pretrainingdistractor', 'end':'newtrial=10'}
NEW_TRIAL       newtrial                    | START
START           distractor                  | 'takedist': PRETRAINING1
      |END
PRETRAINING1    background                  | END
END             background                  | NEW_TRIAL

#THIS IS EXCLUDED AS A REPRESENTATION OF EXP 1 AND REPLACED BY
    EXPERIMENT 1 BELOW.
# Experiment 1, 12x3 trials with token and distractors, delay=15
    minutes
```

```
#@phase {'label':'experiment1', 'end':'newtrial=36'}
#NEW_TRIAL      newtrial                    | START
#START          ('token','distractor')      | 'taketoken': WAIT
            | END
#WAIT           background                  | 15:TOKEN
                      | WAIT
#TOKEN          token                       | 'taketoken':HUMAN
            | END
#HUMAN          human                       | 'givetoken':REWARD
           | END
#REWARD         reward                      | END
#END            background                  | NEW_TRIAL

# Experiment 1, 12x3 trials with token and distractors, three
    choices are carried out in a row before rewards are
# delivered after a delay, delay=15 minutes
@phase {'label':'experiment1', 'end':'newtrial=12'}
NEW_TRIAL       newtrial                    | CHOICE1 CHOICE1
    ('token','distractor')     | 'taketoken': CHOICE2      |
    CHOICE2_1
CHOICE2         ('token','distractor')      | 'taketoken': CHOICE3
          | CHOICE3_1
CHOICE3         ('token','distractor')      | 'taketoken': 3WAIT
           | 2WAIT
CHOICE3_1       ('token','distractor')      | 'taketoken': 2WAIT
           | 1WAIT
CHOICE2_1       ('token','distractor')      | 'taketoken': CHOICE2_2
         | CHOICE2_3
CHOICE2_2       ('token','distractor')      | 'taketoken': 2WAIT
           | 1WAIT
CHOICE2_3       ('token','distractor')      | 'taketoken': 1WAIT
           | END
1WAIT           background                  | 15:1REWARD
                    | 1WAIT
1REWARD         token                       | 'taketoken':HUMAN_1
          | END
HUMAN_1         human                       | 'givetoken':REWARD_1
         | END
REWARD_1        reward                      | END
2WAIT           background                  | 15:2REWARD
                    | 2WAIT
2REWARD         token                       | 'taketoken':HUMAN_2
          | REWARD_3
HUMAN_2         human                       | 'givetoken':REWARD_2
         | END
REWARD_2        reward                      | REWARD_3
REWARD_3        token                       | 'taketoken':HUMAN_3
          | END
HUMAN_3         human                       | 'givetoken':REWARD_4
         | END
REWARD_4        reward                      | END
3WAIT           background                  | 15:3REWARD
                    | 3WAIT
3REWARD         token                       | 'taketoken':HUMAN_4
          | REWARD_6
HUMAN_4         human                       | 'givetoken':REWARD_5
         | REWARD_6
```

```
REWARD_5        reward                        | REWARD_6
REWARD_6        token                         | 'taketoken ':HUMAN_5
        | REWARD_8
HUMAN_5         human                         | 'givetoken ':REWARD_7
        | REWARD_8
REWARD_7        reward                        | REWARD_8
REWARD_8        token                         | 'taketoken ':HUMAN_6
        | END
HUMAN_6         human                         | 'givetoken ':REWARD_9
        | END
REWARD_9        reward                        | END
END              background                    | NEW_TRIAL

# Experiment 3, 14 trials with token, small reward and distractors,
     delay=15 min between taketoken and reward
@phase {'label':'experiment3', 'end':'newtrial=14'}
NEW_TRIAL      newtrial                                   | START
START          ('token','distractor','smallreward')       | 'taketoken ':
    WAIT      |'takesmall ':SMALLREWARD |END
WAIT           background                                 | 15:TOKEN
               | WAIT
TOKEN          token                                      | 'taketoken ':
    HUMAN    | END
HUMAN         human                                      | 'givetoken ':
    REWARD   | END SMALLREWARD    smallreward
                              | END
REWARD         reward                                     | END
END             background                                 | NEW_TRIAL

# Experiment 2, 6 trials with token and distractors delay=17 hours
@phase {'label':'experiment2', 'end':'newtrial=6'}
NEW_TRIAL      newtrial                         | START
START          ('token','distractor')           | 'taketoken ':WAIT
          | END
WAIT           background                       | 1020:TOKEN
                 | WAIT
TOKEN          token                            | 'taketoken ':HUMAN
         | END
HUMAN          human                            | 'givetoken ':REWARD
        | END
REWARD         reward                           | END
END             background                       | NEW_TRIAL

# Experiment 4, 14 trials with token, small reward and distractors,
     no delay between taketoken and reward
@phase {'label':'experiment4', 'end':'newtrial=14'}
NEW_TRIAL      newtrial                                   | START
START          ('token','distractor','smallreward')       | 'taketoken ':
    HUMAN     |'takesmall ':SMALLREWARD |END
HUMAN          human                                      | 'givetoken ':
    REWARD    |END
SMALLREWARD    smallreward                                | END
REWARD         reward                                            | END
END             background                                 | NEW_TRIAL

@run
@figure 'v values '
```

```
@vplot ('token','taketoken') {'steps':'newtrial'}
@vplot ('human','givetoken') {'steps':'newtrial'}
@vplot ('distractor','takedist') {'steps':'newtrial'}
@vplot ('smallreward','takesmall') {'steps':'newtrial'}
@legend #('taketoken','takedist','takesmall') {'steps':'newtrial'}

@figure 'Prob. responding'
@pplot (('token','distractor','smallreward'),'taketoken') {'steps
    ':'newtrial'}
@pplot (('token','distractor','smallreward'),'takedist') {'steps':'
    newtrial'}
@pplot (('token','distractor','smallreward'),'takesmall') {'steps
    ':'newtrial'}
@legend #('token', 'background','distractor')

@figure 'w values'
@wplot ('token') {'steps':'newtrial'}
@wplot ('human') {'steps':'newtrial'}
@wplot ('distractor') {'steps':'newtrial'}
@legend #('token','human')
```

```
( o>
///\
\V_/_
```
